# Resolution of Chemotherapy-Induced Intractable Hiccups Following Osteopathic Manipulative Treatment: A Case Report

**DOI:** 10.7759/cureus.104112

**Published:** 2026-02-23

**Authors:** Pruthvi Sai Chowdary Aluri, Ali Z Ansari, Jasmine L Jorden, Fizza Ahmed, Sahar Hafeez

**Affiliations:** 1 Department of Neurology, Merit Health Wesley, Hattiesburg, USA; 2 Department of Family Medicine, Western Governors University, Salt Lake City, USA; 3 Department of Internal Medicine, William Carey University College of Osteopathic Medicine, Hattiesburg, USA; 4 Department of Neurology, Lake Erie College of Osteopathic Medicine, Bradenton, USA; 5 Department of Pathology and Laboratory Medicine, William Carey University College of Osteopathic Medicine, Hattiesburg, USA

**Keywords:** chemotherapy-induced complications, cisplatin, diaphragmatic doming, hiccup reflex arc, myofascial release, osteopathic manipulative treatment, phrenic nerve inhibition, somatic dysfunction, suboccipital release, thoracic inlet release

## Abstract

Persistent hiccups are a rare but potentially debilitating complication of certain chemotherapeutic agents, particularly cisplatin, and may lead to sleep disruption, nutritional compromise, and decreased quality of life. Chemotherapy-induced hiccups are often refractory to conventional pharmacologic therapies, posing a clinical challenge in oncology care. We present the case of a 67-year-old man with moderately differentiated squamous cell carcinoma of the oropharynx who developed nearly continuous hiccups following his second cycle of cisplatin chemotherapy. Standard pharmacologic interventions, including metoclopramide, gabapentin, and a single dose of intravenous chlorpromazine, provided only transient or minimal relief. Osteopathic evaluation identified somatic dysfunctions involving the mid-cervical spine (C3-C5), upper thoracic segments, thoracic inlet, an elevated left first rib, restricted right hemidiaphragmatic excursion, and cranial base restrictions. These findings were considered potential contributors to altered diaphragmatic mechanics and dysregulation of the hiccup reflex arc. A single session of osteopathic manipulative treatment (OMT) directed at these regions was associated with rapid and complete resolution of hiccups, with sustained improvement in sleep, oral intake, and overall functional status. The patient tolerated subsequent chemotherapy cycles without recurrence of symptoms and did not require additional pharmacologic or osteopathic interventions. While causal inference cannot be established from a single case, this report suggests that OMT may serve as a safe adjunctive therapy for refractory chemotherapy-induced hiccups, potentially through effects on diaphragmatic motion, myofascial tension, and autonomic regulation.

## Introduction

Persistent hiccups, characterized by involuntary, repetitive contractions of the diaphragm and intercostal muscles followed by abrupt closure of the glottis, represent a rare but clinically significant condition that can substantially impact patient well-being [1]. While transient hiccups are common, benign, and self-limiting, persistent (>48 hours) or intractable (>1 month) hiccups are associated with a wide range of complications, including sleep disturbance, fatigue, dehydration, malnutrition, weight loss, and impaired overall functional status [2]. Etiologies are diverse and encompass central nervous system disorders, metabolic and electrolyte disturbances, gastrointestinal pathology, thoracic or mediastinal disease, and medication-induced causes [3]. Among iatrogenic factors, chemotherapy has emerged as an important, albeit uncommon, contributor to persistent hiccups. Cisplatin, a platinum-based chemotherapeutic agent widely used in head and neck, lung, and gynecologic malignancies, has been particularly implicated [4]. These episodes can be especially burdensome in oncology patients, who are already vulnerable to fatigue, poor oral intake, and impaired quality of life, and may complicate adherence to planned chemotherapy regimens.

Management of persistent hiccups presents a clinical challenge due to the limited efficacy of conventional pharmacologic therapies. Standard treatment options, including dopamine antagonists such as metoclopramide or chlorpromazine, gamma-aminobutyric acid modulators such as gabapentin or baclofen, and antacids or proton pump inhibitors when gastrointestinal irritation is suspected, often produce incomplete or transient relief [5]. Non-pharmacologic interventions, such as vagal maneuvers or diaphragmatic breathing exercises, are typically reserved for mild or intermittent cases and are frequently insufficient for severe or intractable presentations [6]. Osteopathic manipulative treatment (OMT) offers a complementary, structure- and function-based approach to management by addressing musculoskeletal, fascial, and autonomic dysfunctions that may contribute to diaphragmatic hyperexcitability and dysregulation of the hiccup reflex arc [7]. Techniques targeting the cervical and thoracic spine, thoracic inlet, ribs, diaphragm, and cranial base have been proposed as adjunctive strategies aimed at improving respiratory mechanics and supporting neuromuscular function in cases of persistent hiccups [8].

## Case presentation

A 67-year-old Caucasian man, a retired factory machinist, was referred for osteopathic evaluation due to persistent and debilitating hiccups that developed in close temporal association with chemotherapy. He had been diagnosed with moderately differentiated squamous cell carcinoma of the oropharynx, clinically staged as T2N1M0 based on imaging studies and confirmatory tissue biopsy. At the time of evaluation, he was undergoing definitive treatment with a cisplatin-based chemotherapy regimen, consisting of cisplatin administered at a dose of 100 mg/m² every 21 days. The first cycle of chemotherapy was completed without complication, and the patient did not experience hiccups, nausea, or any other treatment-related adverse effects, indicating initial good tolerance of the regimen. The development of persistent hiccups following the second cycle prompted further clinical attention due to the severity and impact of symptoms on his daily functioning.

His past medical history was significant for well-controlled essential hypertension managed with lisinopril and moderate chronic obstructive pulmonary disease (COPD) managed with an inhaled long-acting bronchodilator. He had a remote 20 pack-year smoking history but had ceased tobacco use approximately 10 years prior. He also reported chronic opioid use for cancer-related pain management. He denied gastroesophageal reflux disease, peptic ulcer disease, esophageal motility disorders, cervical or thoracic spine pathology, neurologic disease, or prior episodes of persistent or intractable hiccups. There was no history of diabetes mellitus, chronic kidney disease, or electrolyte abnormalities. At baseline, the patient was fully independent in all activities of daily living. His height was 178 cm, weight was 82 kg, and body mass index was 25.9 kg/m².

Approximately 48 hours after completion of the second chemotherapy cycle, the patient began experiencing intermittent hiccups. Initially brief and sporadic, the episodes increased progressively in both frequency and intensity over the subsequent 48 hours. By the third day after infusion, hiccups had become nearly continuous during waking hours, occurring in repetitive clusters with only brief periods of relief. The episodes frequently disrupted sleep, awakening the patient multiple times each night and preventing sustained rest. As the hiccups persisted, the patient experienced progressive fatigue, discomfort in the chest wall due to repetitive diaphragmatic contractions, and difficulty maintaining adequate oral intake. He reported early satiety and nausea exacerbated by hiccup episodes, as well as dysphagia related to ongoing oropharyngeal pain from his malignancy. Nursing documentation confirmed frequent hiccups occurring every 20-30 seconds. Mild dehydration and subjective weakness developed over the following days, although he remained hemodynamically stable and afebrile.

Standard pharmacologic therapies were initiated sequentially. The patient received oral metoclopramide and gabapentin without sustained improvement. A single intravenous dose of chlorpromazine resulted in only transient symptom reduction. Baclofen was considered but deferred due to concerns for excessive sedation and potential respiratory compromise in the context of concurrent opioid use for cancer-related pain and underlying COPD. Laboratory evaluation revealed no significant electrolyte abnormalities. Given the temporal relationship with chemotherapy, absence of neurologic deficits, and lack of alternative pathology, the hiccups were attributed to chemotherapy-induced intractable hiccups.

Due to refractory symptoms and significant functional impairment, an osteopathic consultation was requested on day 5 following chemotherapy. At evaluation, the patient was alert, oriented, and cooperative but visibly fatigued. Recurrent hiccup episodes were observed in clusters every 20-30 seconds. Vital signs were stable, and oxygen saturation was 96% on room air. Cardiopulmonary examination revealed clear lung fields and normal heart sounds without murmurs, rubs, or gallops. Abdominal examination was unremarkable, except for mild tenderness along the right subcostal margin. Neurologic evaluation was non-focal, with intact cranial nerves, normal strength and tone, symmetric reflexes, and no sensory deficits. No signs of increased intracranial pressure or central nervous system pathology were noted.

A comprehensive osteopathic structural examination was performed with particular attention to regions influencing respiratory mechanics, diaphragmatic innervation, and autonomic regulation. Examination of the cervical spine revealed pronounced bilateral paraspinal hypertonicity, most notably within the anterior scalene musculature. Segmental motion testing demonstrated somatic dysfunction of the mid-cervical spine at levels C3 through C5, characterized by a flexed positional preference with rotation and sidebending to the right, along with restricted motion to the left. These segments correspond anatomically to the spinal nerve roots contributing to the phrenic nerve. Evaluation of the thoracic spine identified somatic dysfunction spanning levels T2 through T6, characterized by a neutral positional preference with sidebending to the right and rotation to the left, accompanied by left-sided paraspinal hypertonicity and tissue texture changes, and restricted segmental motion. Thoracic cage compliance was reduced on the left, with asymmetric expansion observed during respiratory excursions. Palpation of the ribs revealed an elevated left first rib with restriction of exhalation, associated with myofascial tension involving the anterior scalene and pectoralis minor muscles. The thoracic inlet demonstrated restricted motion with rotation to the right and sidebending to the left, along with fascial congestion, suggesting impaired lymphatic and neurovascular flow through this region. Assessment of the diaphragm revealed restricted excursion of the right hemidiaphragm during both passive and active respiration, accompanied by palpable myofascial tension along the right costal margin. Cranial evaluation demonstrated somatic dysfunction of the occipitoatlantal joint characterized by a flexed position with right rotation and left sidebending, with decreased left rotational motion, palpable tension across the occipital and temporal regions, and a diminished, asymmetric cranial rhythmic impulse. A summary of these findings is presented in Table 1.

**Table 1:** Somatic dysfunctions and structural observations identified during osteopathic evaluation

Region	Somatic dysfunction description
Cervical spine (C3-C5)	Somatic dysfunction characterized by a flexed positional preference with rotation and sidebending to the right, associated with bilateral paraspinal hypertonicity and restricted motion to the left
Anterior cervical soft tissues	Hypertonicity of the anterior scalene musculature
Thoracic spine (T2-T6)	Somatic dysfunction characterized by a neutral positional preference with sidebending to the right and rotation to the left, associated with left-sided paraspinal hypertonicity and tissue texture changes, and restricted segmental motion
Thoracic cage	Decreased compliance on the left with asymmetric respiratory excursion
Rib 1 (left)	Elevated left first rib with restriction of exhalation
Upper thoracic soft tissues	Myofascial tension involving the anterior scalene and pectoralis minor muscles
Thoracic inlet	Restricted motion with rotation to the right and sidebending to the left, with associated fascial congestion
Diaphragm	Restricted excursion of the right hemidiaphragm with palpable myofascial tension along the right costal margin
Occipitoatlantal joint	Somatic dysfunction characterized by a flexed position with right rotation and left sidebending, associated with decreased left rotation
Cranial	Diminished and asymmetric cranial rhythmic impulse with palpable occipital and temporal tension

A single session of OMT was administered with the patient positioned in supine and seated postures as clinically appropriate. Treatment was directed toward reducing myofascial tension, restoring the physiologic motion of the diaphragm and thoracic cage, and modulating autonomic tone associated with the hiccup reflex arc. Given the patient’s ongoing oncologic treatment, fatigue, and overall clinical status, techniques were applied gently and primarily using indirect approaches. Initial treatment focused on the cranial base and upper cervical region through soft tissue and suboccipital decompression techniques to reduce tension at the occipitoatlantal junction and facilitate parasympathetic activity mediated by the vagus nerve. Gentle myofascial release was then applied to the anterior scalene musculature to reduce tension affecting the cervical spinal segments at levels C3 through C5, corresponding to the course of the phrenic nerve. Subsequent treatment addressed the upper thoracic region using rib raising techniques to decrease sympathetic nervous system hyperactivity and improve thoracic segmental mobility. This was followed by myofascial release of the thoracic inlet to reduce fascial congestion and enhance lymphatic and neurovascular flow through this region. Diaphragmatic doming techniques were then applied to improve diaphragmatic excursion and normalize respiratory mechanics. Balanced membranous tension cranial techniques were performed at the conclusion of the treatment session to address residual restrictions at the cranial base and support overall autonomic regulation. A summary of the osteopathic manipulative techniques utilized during this session is presented in Table 2.

**Table 2:** OMT techniques applied during a single treatment session, organized by anatomic region with associated therapeutic rationale and intended physiologic effects OMT: osteopathic manipulative treatment

Anatomic region treated	Osteopathic manipulative technique applied	Therapeutic rationale and intended physiologic effect
Cranial base and upper cervical region	Suboccipital decompression and soft tissue techniques	Reduce tension at the occipitoatlantal junction and facilitate parasympathetic activity via vagal nerve pathways
Anterior cervical region (C3-C5)	Gentle myofascial release of the anterior scalene musculature	Decrease tension affecting the cervical spinal segments associated with the phrenic nerve and reduce nerve excitability
Upper thoracic spine	Rib raising techniques	Decrease sympathetic nervous system hyperactivity and improve thoracic segmental mobility
Thoracic inlet	Myofascial release of the thoracic inlet	Reduce fascial congestion and enhance lymphatic and neurovascular flow
Diaphragm	Diaphragmatic doming techniques	Improve diaphragmatic excursion and normalize respiratory mechanics
Cranial	Balanced membranous tension cranial techniques	Address residual cranial base restrictions and promote overall autonomic balance

Immediately following the completion of the OMT session, the patient experienced a rapid clinical response. Within minutes, the frequency of hiccups decreased substantially and resolved during the immediate post-treatment observation period. This was confirmed through direct observation, during which the patient was able to speak continuously and take slow, deep breaths without interruption or diaphragmatic spasm. He reported an immediate sensation of relaxation across the anterior neck, chest, and upper abdomen, describing his breathing as deeper, more effortless, and free from the persistent urge to hiccup. He also noted relief from chest wall discomfort that had developed secondary to repetitive diaphragmatic contractions. The patient was closely monitored for approximately 20 minutes following the intervention, during which no recurrence of hiccups or adverse effects were observed.

Later that same day, a follow-up assessment by the oncology team confirmed the continued absence of hiccups. The patient reported improved tolerance of oral intake and described a profound sense of overall physical relief compared with the preceding days. That evening, he was able to sleep uninterrupted for several hours for the first time since symptom onset, which he identified as a meaningful improvement in his quality of life. He denied any recurrence of hiccups overnight and reported no nausea, dizziness, or other adverse effects attributable to the osteopathic intervention.

At 48-hour follow-up, the patient remained completely free of hiccups. He described sustained improvements in sleep quality, resolution of fatigue and irritability associated with prior sleep disruption, and notable enhancement of oral intake and hydration. He also reported increased energy levels and decreased chest wall soreness, attributing these changes to the cessation of repetitive diaphragmatic contractions. No new neurologic, gastrointestinal, or respiratory symptoms were reported. At one-week follow-up, the patient continued to demonstrate complete and sustained remission of hiccups without any breakthrough episodes and had not required additional pharmacologic therapy. His functional status had returned to baseline, and physical examination remained unremarkable. Importantly, the patient was able to proceed with subsequent cycles of cisplatin-based chemotherapy without recurrence of hiccups. While sustained symptom remission was observed, the contribution of nonspecific, contextual, or placebo-related effects cannot be excluded, and no additional OMT sessions or medical interventions were required during follow-up.

## Discussion

The clinical course of this patient’s persistent hiccups suggests that the symptom burden he experienced was not simply an isolated adverse effect of chemotherapy, but rather the result of a complex interaction between chemotherapy-induced neural sensitization and preexisting mechanical and autonomic influences. The escalation of hiccups following the second cycle of cisplatin, rather than the first, raises the possibility of cumulative neurotoxicity or progressive sensitization of the hiccup reflex arc. Cisplatin is known to affect peripheral and central neural pathways, and in this setting, it is reasonable to consider that the medication may have lowered the threshold for reflex activation [9]. Once this threshold was reduced, even modest mechanical or autonomic disturbances may have been sufficient to perpetuate continuous diaphragmatic contractions. This framework helps explain why the hiccups rapidly intensified and became resistant to pharmacologic suppression, despite the absence of metabolic derangements or structural neurologic disease.

The failure of multiple standard medical therapies further supports the notion that the dominant drivers of this patient’s symptoms were not purely neurochemical. Medications such as metoclopramide, gabapentin, and chlorpromazine primarily target central neurotransmitter pathways, yet their limited and transient effect in this patient suggests that peripheral afferent input and mechanical reinforcement of the reflex arc remained unaddressed [10]. In this context, ongoing afferent signaling from the diaphragm, cervical spine, or thoracic structures may have continuously reactivated the reflex, overwhelming central inhibition. The persistence of hiccups despite sedation and neuromodulation emphasizes the importance of considering structural contributors, particularly when symptoms are severe, continuous, and temporally related to physiologic stressors such as chemotherapy.

The osteopathic structural findings identified during evaluation offer a coherent explanation for how this reflex may have been perpetuated. Dysfunction within the anterior scalene musculature at the C3 through C5 levels is particularly significant given the anatomic origin of the phrenic nerve. Hypertonicity and fascial restriction in this region may increase mechanical irritation or compressive stress on the nerve, heightening its excitability [11]. In a patient already experiencing chemotherapy-related neural sensitization, this localized dysfunction may have played a disproportionate role in sustaining diaphragmatic spasms. The fact that cervical motion was restricted further suggests altered proprioceptive input and sustained muscle guarding, both of which can amplify reflexive motor activity.

Thoracic and rib dysfunctions likely compounded this effect by altering respiratory mechanics and reinforcing abnormal diaphragmatic motion. Restricted thoracic cage compliance and asymmetric rib motion can prevent smooth diaphragmatic excursion, encouraging abrupt or repetitive contractions rather than coordinated respiration [12]. In this patient, decreased thoracic mobility may have created a mechanical environment in which each diaphragmatic contraction further reinforced the next, establishing a self-sustaining cycle. Thoracic inlet restriction adds another layer of complexity, as impaired fascial mobility in this region can affect neurovascular flow and autonomic signaling between the head, neck, and thorax [13]. In an already stressed system, these restrictions may have contributed to autonomic imbalance, further lowering the threshold for reflex activation.

The diaphragmatic findings themselves are particularly important when reflecting on the patient’s clinical response. Decreased excursion of the right hemidiaphragm and palpable myofascial restriction along the costal margins suggest that the diaphragm was functioning under mechanical constraint. When diaphragmatic motion is restricted, afferent feedback from muscle spindles and mechanoreceptors may become exaggerated, feeding into the hiccup reflex arc [14]. This persistent afferent signaling could explain why the hiccups were so frequent and resistant to interruption. Addressing this restriction directly through osteopathic techniques may therefore have removed a critical driver of ongoing reflex activation.

Cranial findings, particularly restriction at the occipitoatlantal junction, offer insight into the autonomic component of the patient’s symptoms. This region is anatomically and functionally significant due to its relationship with the vagus nerve and parasympathetic regulation [15]. Tension or restriction at the cranial base may influence vagal tone, potentially increasing excitability within the medullary centers involved in the hiccup reflex [16]. In this patient, cranial restrictions may have contributed to sustained autonomic imbalance, amplifying the reflex response initiated by peripheral inputs. The diminished and asymmetric cranial rhythmic impulse further suggests a system under strain, with reduced adaptability and impaired self-regulation.

The sustained absence of hiccups during subsequent chemotherapy cycles provides additional insight into the significance of the intervention. If the hiccups had been driven solely by cisplatin toxicity, recurrence would have been expected with continued exposure. Instead, the durability of symptom resolution suggests that the osteopathic intervention addressed underlying vulnerabilities that predisposed the patient to reflex hyperexcitability. By restoring structural balance and improving physiologic motion, the treatment may have increased the patient’s resilience to subsequent chemotherapeutic stressors. This observation reinforces the concept that structural and functional optimization can have lasting effects, even in the presence of ongoing systemic challenges.

Beyond symptom resolution, the broader clinical impact of this intervention is noteworthy. Improvement in sleep, oral intake, hydration, and overall comfort reflects meaningful restoration of physiologic function and quality of life. In oncology patients, these outcomes are particularly important, as they influence treatment tolerance, recovery, and overall well-being. The absence of adverse effects and the non-pharmacologic nature of the intervention further highlight its suitability in patients with complex medical profiles and polypharmacy. Taken together, this case illustrates how persistent hiccups can arise from a convergence of chemotherapeutic effects and somatic dysfunction, and how targeted osteopathic intervention can disrupt this convergence in a clinically meaningful way. Although the clinical outcome in this case was favorable, the findings are inherently limited by the single-patient design. Causal inference cannot be established, and factors such as natural symptom fluctuation, nonspecific therapeutic effects, and contextual influences cannot be excluded. The observations presented should therefore be interpreted as hypothesis-generating rather than confirmatory and are not intended to imply generalizability beyond this individual case.

## Conclusions

Persistent hiccups represent a rare but clinically significant complication in patients undergoing chemotherapy and may result in substantial functional impairment when refractory to standard medical therapy. This case suggests that chemotherapy-associated hiccups may arise from an interplay between neural sensitization and underlying somatic dysfunction, rather than from pharmacologic effects alone. The patient’s rapid and sustained response following a single session of OMT highlights the potential clinical relevance of identifying structural, myofascial, and autonomic contributors to symptom persistence. Although causal inference cannot be established from a single case, osteopathic intervention in this instance was temporally associated with meaningful improvements in diaphragmatic motion, sleep, oral intake, and overall quality of life, without the need for additional medications. These findings support further exploration of OMT as a potential adjunctive approach for refractory hiccups in oncology patients. As a single case report, this observation should be considered hypothesis-generating and intended to encourage additional study rather than to establish definitive therapeutic efficacy.
